# Role of Multifunctional Cytoskeletal Filaments in *Coronaviridae* Infections: Therapeutic Opportunities for COVID-19 in a Nutshell

**DOI:** 10.3390/cells10071818

**Published:** 2021-07-19

**Authors:** Victor Norris, Judit Ovádi

**Affiliations:** 1 Laboratory of Microbiology Signals and Microenvironment, University of Rouen, 76821 Mont Saint Aignan, France; victor.norris@univ-rouen.fr; 2 Institute of Enzymology, Research Centre for Natural Sciences, ELKH 1117 Budapest, Hungary

**Keywords:** cytoskeleton, filaments, SARS-CoV-2, COVID-19, drug targets

## Abstract

A novel coronavirus discovered in 2019 is a new strain of the *Coronaviridae* family (CoVs) that had not been previously identified in humans. It is known as SARS-CoV-2 for *Severe Acute Respiratory Syndrome Coronavirus-2*, whilst COVID-19 is the name of the disease associated with the virus. SARS-CoV-2 emerged over one year ago and still haunts the human community throughout the world, causing both healthcare and socioeconomic problems. SARS-CoV-2 is spreading with many uncertainties about treatment and prevention: the data available are limited and there are few randomized controlled trial data on the efficacy of antiviral or immunomodulatory agents. SARS-CoV-2 and its mutants are considered as unique within the *Coronaviridae* family insofar as they spread rapidly and can have severe effects on health. Although the scientific world has been succeeding in developing vaccines and medicines to combat COVID-19, the appearance and the spread of new, more aggressive mutants are posing extra problems for treatment. Nevertheless, our understanding of pandemics is increasing significantly due to this outbreak and is leading to the development of many different pharmacological, immunological and other treatments. This Review focuses on a subset of COVID-19 research, primarily the cytoskeleton-related physiological and pathological processes in which coronaviruses such as SARS-CoV-2 are intimately involved. The discovery of the exact mechanisms of the subversion of host cells by SARS-CoV-2 is critical to the validation of specific drug targets and effective treatments.

## 1. Overview: Coronaviruses and the Cytoskeleton

**Figure 1:**
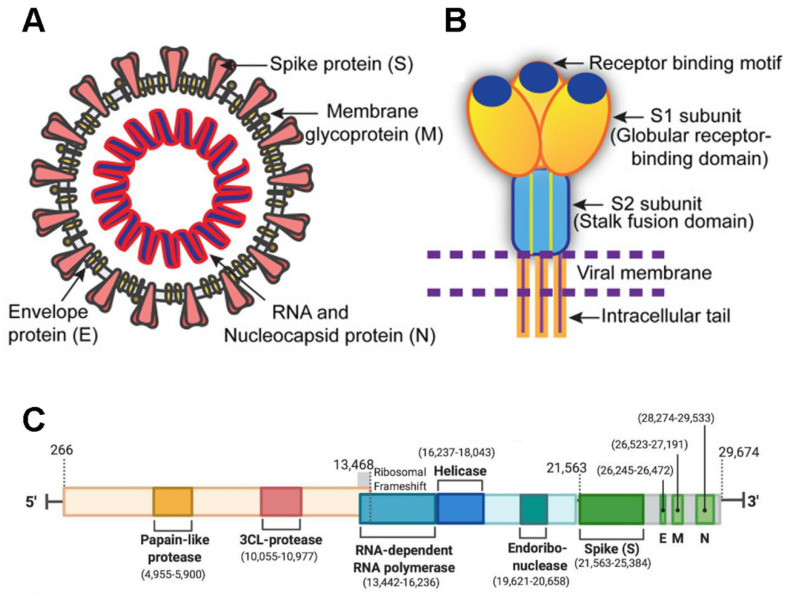
Structure of SARS-CoV-2. (**A**) The virus with the spike (S), envelope (E), membrane (M), and nucleocapsid (N) proteins are displayed. (**B**) The ACE-2 receptor. (**C**) Genomic organization. The orange-brown and the blue-green sections correspond to ORF1a and ORF1b, respectively. The illustrations are adapted from [2,3].

SARS-CoV-2 is a member of the Coronaviridae family and has a large 29,903-nucleotide, positive-strand RNA genome [1]. The genome organization of SARS-CoV-2 is similar to other CoVs such as HCoV-OC43, MERS-CoV, SARS-CoV, SARS-CoV-2, HCoV-229E, and HCoV-NL63. One third of its genome encodes accessory and structural proteins, which includes the spike (S), envelope (E), membrane (M), and nucleocapsid (N) proteins [2,3] (green blocks in Figure 1); the other two thirds of the genome are occupied by two large overlapping open reading frames (ORF1a and ORF1b) that are translated into polyproteins and are processed to generate 16 non-structural proteins. A ribosomal frameshift is located between ORF1a and ORF1b. The non-structural proteins include papain-like protease, 3CL-protease, RNA-dependent RNA polymerase, helicase, endoribonuclease, and viroporins.

**Figure 2:**
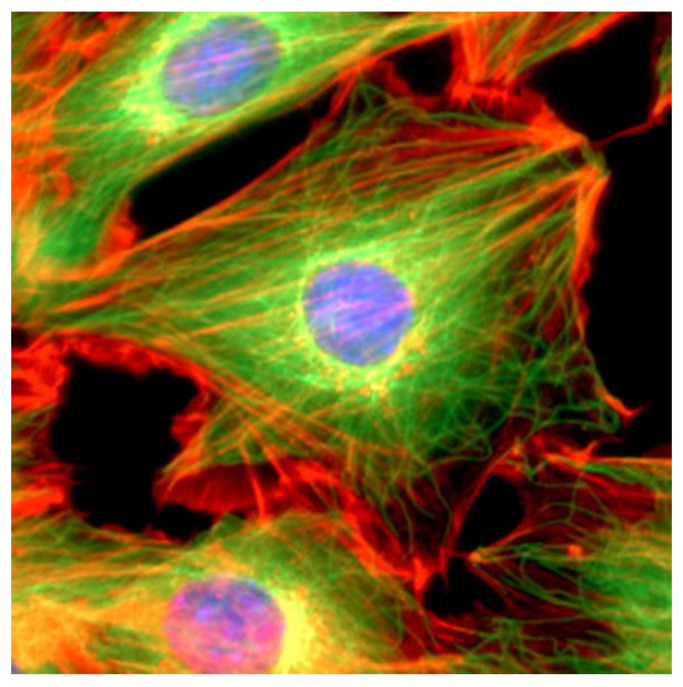
Immunofluorescence image of a 3T3 mouse fibroblast cell showing nuclear DNA (blue), actin microfilaments (red), and alpha-tubulin (green). The illustration is adapted from [6].

The cytoskeleton, which plays a key role in several viral processes, is an intricate network of filaments running through the cytoplasm that helps cells to maintain their shape and internal organization (Figure 2). This is achieved via the dynamics of the actin filaments (AFs), microtubule filaments (MFs), and intermediate filaments (IFs) that constitute the cytoskeletal network [4,5]; in particular, the cytoskeleton provides the mechanical support that enables cells to perform many vital functions.

These functions include cargo transport, signal transduction, and the control of cell shape, movement, and division. Recent studies have shown the importance of the interactions between coronaviruses and cytoskeletal filaments. The entry of the virus involves IFs and MFs, which is followed by the use of MFs for transportation to replication and assembly sites, and the harnessing of the polymerization of AFs to force release [1].

## 2. Cytoskeletal Transport of Influenza Viruses

**Figure 3:**
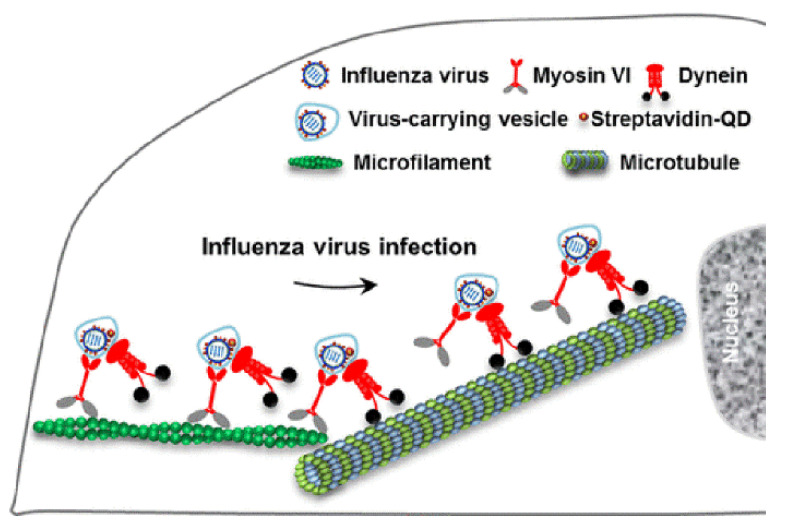
Transport model of influenza virus from AFs to MTs; the illustration is reprinted (adapted) with permission from [9]. Copyright {2017} American Chemical Society.

Viral homologs of host proteins can mimic fundamental cell process during the course of the viral life cycle [7]. The well-characterized influenza virus is often used as a model system for understanding viral infections [8]. When infecting host cells, the virus must move along AFs at the cell periphery and then move along MFs through the cytosol to reach the perinuclear region for genome release [9]. Within the host cells, myosin VI (MyoVI) and dynein are responsible for virus transport on the AFs and MFs with the two motor proteins being attached to the same virus-carrying vesicle (Figure 3). MyoVI drives viruses along AFs with dynein as a passenger on the vesicle, and then dynein drives the viruses along MFs with MyoVI as a passenger. It has been revealed that the “driver switchover” mechanism from AFs to MFs enables the successful transport of the virus within the host cell [9].

## 3. Microtubule Filaments and Coronaviruses

In many cell types, cytoplasmic dynein motors transport cargoes in a retrograde manner toward the minus end of MTs, which are frequently anchored at the MicroTubule Organizing Center (MTOC) [10]. The long-range transport of the coronaviruses to and from the cell periphery is mediated by dynein and kinesin on the MFs, while the actin and myosin filaments mediate the short-range transport [11].

MFs are involved at the entry stage where the cytoplasmic tail of the S protein binds to tubulin (Figure 1) and where dynamin, an MT-organizing protein [12], is important for the internalization step [13].

CoV infections of cells may stimulate the formation of autophagosomes, which have double membranes (see Section 6); this formation is facilitated by MFs on which their subsequent movement and fusion depend [14]. In fact, CoV infections are considered to entail processes closely associated with autophagy, which may even promote CoV infection and replication ([15] and references therein). However, the interplay between the autophagy machinery and the CoVs including the SARS-CoV-2 is unclear so far [16].

The trafficking of viruses on the MT network by dynein and kinesin-1 motors plays an important role in the replication and spread of many viruses, as supported by excellent studies carried out with *Porcine Epidemic Diarrhea Virus* (PEDV), a member of the Coronavirus family [17]. Using a single-virus tracking technique, the molecular mechanisms of the transport of PEDV have revealed the involvement of this trafficking machinery in the fusion of the virus with the membrane and in its accumulation in the perinuclear region. The dynamically monitored intracellular transport displayed different mechanisms such as unidirectional movements toward MT plus/minus ends as well as bidirectional movements along different MTs. The findings of these studies have greatly contributed to our understanding of the pathogenesis of CoVs.

Finally, in the context of MFs, cilia should also be considered. Cilia are composite structures based on MTs and present on the cell surface [18]. Ciliopathies are associated with a wide range of clinical features that include chronic respiratory problems. It is therefore significant that structural damage to the respiratory epithelium and abnormal ciliary function are typical pathological symptoms of CoV infection with different CoVs causing cilia loss (via changes to the structure of MFs) and/or severe damage in the upper respiratory tract and lung [18].

## 4. Actin Filaments and Coronaviruses

After binding to the host cell, viruses can use the depolymerization of AFs to “surf” to their entry sites where further dynamic rearrangements of AFs are involved in virus internalization [1]. On one hand, cofilin, a host protein that promotes AF depolymerization, is phosphorylated by some coronaviruses to inhibit this depolymerization and assist with entry [19]. On the other hand, ezrin, a host protein that links the membrane to the actin cytoskeleton, can bind to the C-terminus of the Spike protein and inhibit the entry and fusion of SARS-CoV [20]. In the case of antibody binding to Feline Infectious Peritonitis Virus-infected monocytes, internalization is initiated and driven by Myo I and Myosin Light Chain Kinase, whilst subsequent passage through the cortical actin barrier involves Myo VI [11]. At the later stage of the synthesis of the viral proteins and genomes, AFs retract from the plasma membrane to form a ring associated with the nucleus and to bind to virus particles near the nuclear membrane [1]. The disruption of AF dynamics counteracts the actin ring formation and virus replication. It should be noted that actin rearrangements can result from the action of the N protein of SARS-CoV, which induces the p38 mitogen-activated protein kinase cascade [21]. The viral structural proteins move to the ER-Golgi intermediate compartment where, at least in one coronavirus, an AF-crosslinking protein, filamin A, interacts with the Spike protein [22]. Finally, interaction between β-actin and the M protein is essential for the assembly and budding of Infectious Bronchitis Virus [23], whilst the thickening of AFs below the cell surface is proposed to provide the bending force to extrude SARS-CoV particles [24].

## 5. IFs and Coronaviruses

The IF networks of mammalian cells form highly dynamic linkages between the cell surface and the nucleus that undergo functionally significant changes in their organization during various cellular processes [25]. Their major physiological functions include scaffolding, membrane trafficking, and signal transduction. Consequently, abnormalities of IFs lead to pathogenesis. Studies of the dynamic characteristics of the interactions between the cytoskeleton filaments and coronaviruses such as SARS-CoV have shown that vimentin, a component of IFs, acts as a co-receptor for the entry of the virus [1]. Vimentin has an affinity for gangliosides [26] to which SARS-CoV-2 binds via its N-terminal domain [27] and, significantly, lipid rafts including gangliosides are enriched in the ACE-2 receptor [28]. The angiotensin-converting enzyme 2 (ACE2) is the principal receptor on the host cell surface. At the later stage of coronavirus replication, vimentin, which also binds to the N protein, is essential [29].

## 6. Interconnection of Autophagy and CoV Infection

Virus–host interactions involve cellular degradative pathways such as macroautophagy/autophagy [30]. Autophagy, the major pathway, entails the engulfment of the cargo/vesicles by a double-membrane phagophore to form an autophagosome via the actions of microtubule-associated protein light chain 3 (LC3B) and the sequestosome (SQSTM1/p62). The autophagosome then fuses with the lysosome to form an autolysosome, which provides an acidic milieu for the pH-dependent degradation of proteins, lipids, nucleic acids, and vesicles. Autophagy enables cells to degrade and recycle damaged organelles and proteins; its destruction leads to different diseases.

Coronaviruses as intracellular pathogens without machinery for self-replication have a complex relationship with the autophagy of the host cells in producing viral particles and escaping host defenses. Evidence exists for both coronavirus-induced autophagy and coronavirus-induced arrest of autophagy. Indeed, it has been reported that chloroquine (CQ), an autophagy inhibitor, can counteract coronavirus infection. However, it has also been reported that autophagy can be either detrimental or beneficial to viral replication and maturation [30]; in the latter case, it was found that the interaction of coronaviruses with autophagy could actually increase the replication of the virus.

Interestingly, the ER-derived double-membrane vesicles formed by coronaviruses in the host cytoplasm are so similar to autophagosomes that it has been suggested that coronaviruses mimic the cellular autophagy pathway [31]. Such structural mimicry would be important since the double-membrane vesicles (DMVs) serve as sites for the replication of the CoV genome [30]. By orchestrating overall antiviral defenses, autophagy could prevent the infection of the host as well as the “cytokine storm,” which should be controlled in the case of COVID-19 patients [1].

## 7. Therapeutic Approaches to COVID-19 Based on the Cytoskeleton

The active involvement of the cytoskeleton in the hijacking of the cells infected by the viruses is confirmed by the fact that targeting the MT cytoskeleton with MT inhibitors reduces the viral load. Thus, a potential treatment of coronavirus-infected individuals with MT-targeting drugs might be effective. A number of drugs used in cancer therapy alter the dynamics and stability of MTs. While the *vinca* alkaloids result in the disassembly of the MT network, paclitaxel stabilizes it; both agents therefore prevent chromosome segregation and abolish cell division. Such effects are of great importance in chemotherapy medication, as they would be to a MT-based treatment for virus infection/transmission. The effect of colchicine, which inhibits microtubule polymerization, on the efficacy and safety outcomes of COVID-19 patients has been explored [32]. If its effectivity is proved, it would be a significant milestone in the management of COVID-19, a disease with limited available therapeutic options. This possibility needs to be investigated.

CoV infection induces the phosphorylation of the Microtubule Associated Protein, tau, via a glycogen synthase kinase-3b-dependent mechanism; this disrupts the MT-stabilizing capacity of tau and results in brain damage that is related to neurodegenerative diseases such as *tauopathy* [33]. In addition, a parallel can be drawn between CoV infection and the progression of demyelinating diseases such as multiple sclerosis, which is also correlated with MF-dependent transport processes.

In the case of AFs, a compound directed against ezrin could be used to inhibit SARS-CoV-2 entry. Ezrin peptides have been used to treat infections that include HIV-1, hepatitis C virus, human papillomavirus, herpes simplex I and II, acute viral respiratory infection and, in particular, in the inhibition of inflammation in viral pneumonia, a serious complication that can occur in COVID-19 (for references, see [34]).

Cytokines and pro-inflammatory mediators implicated in respiratory viral infections, which include bradykinin and tumour necrosis factor-alpha (TNFα), disrupt the actin cytoskeleton. In COVID-19-related lung disease, which is a leading cause of death from this disease, these cytokines play a major role. First, for example, bradykinin is associated with a calcium-dependent actin reorganization and an increased permeability [35], whilst ACE2 internalization due to SARS-CoV-2 infection is believed to create an imbalance in the Kinin–Kallikrein system, and the consequent overactivation of the pathway involving bradykinin and related peptides results in an increase in inflammation [36]. Second, TNFα acts via Rho-kinase to disintegrate the endothelial and epithelial cytoskeleton and hence damage the intercellular barrier and flood the interstitial spaces with fluid ([37] and references therein). Disruption of the actin cytoskeleton disrupts the microdomain organization of the plasma membrane and hence the interaction between phosphodiesterase type 3A (PDE3A) and the cystic fibrosis transmembrane conductance regulator (CFTR) channel; this, in turn, leads to a disruption of compartmentalized cAMP signalling and to reduced secretion [38]. It is therefore reasonable to suppose that restoring the cytoskeleton-dependent integrity of the cell barriers within the lung would constitute a valuable approach to treating COVID-19 lung disease.

In line with the above, colchicine (see above) attenuates the inflammatory response by interfering with pathways that include the TNFα-induced nuclear factor κΒ (NF-κΒ) pathway [39]. The PDE3-inhibitors, milrinone and enoximone, are reported to help treat respiratory failure in patients with severe SARS-CoV-2 pneumonia [40]. PDE3-inhibitors prevent microvascular leakage (via a normalization of the cytoskeleton by increasing intracellular cAMP) and increase the ciliary beat frequency of the epithelial cells within the respiratory tract ([40] and references therein); moreover, the anti-inflammatory properties of PDE3-inhibitors may help prevent the cytokine storm [41]. Finally, dexamethasone has been shown to preserve the intestinal mucosal barrier and to shorten the length of hospitalization of COVID-19 patients [42].

## 8. Other Therapeutic Approaches to COVID-19

Although the transmission of severe acute respiratory syndromes caused by the viruses in human populations is known to lead to massive health and socioeconomic crises, the recently discovered coronavirus, SARS-CoV-2, has been considered to be unique in its fast, worldwide spread and the severity of its effects on health. The relationship between viral infection and autophagy is known as virophagy [43]. It has been proposed that SARS-CoV-2 hijacks virophagy to create a virus factory for replication, immune escape, exocytosis, and ultimately the inflammatory storm associated with the most severe COVID-19 cases and, consistent with this, pharmacological approaches based on autophagy are being tested in over half of clinical trials on patients with COVID-19 [43].

CQ and hydroxychloroquine (HCQ) have been used to treat malaria for many years; recently, they have been used for treatment and prevention of COVID-19 [44]. In fact, CQ is a well-known inhibitor of autophagosome formation and hence of autophagy maturation. As regards its involvement in the viral pathway, it has been proposed to act on both cellular entry and exit. These drugs alter intracellular pH, and may induce ER stress, causing misformation of essential viral proteins. Recently, it has been shown that HCQ, which has a strong affinity for the sialic acid constituents of glycoproteins and gangliosides, can bind to lipid raft gangliosides and neutralize virus binding and infection [27]. Remdesivir, or GS-5734, is a broad-spectrum, antiviral, phosphoramidate pro-drug, which is involved in the incorporation of ribonucleotides into nascent viral RNA chains. It confuses the viral, RNA-dependent, RNA polymerase and delays, or prematurely terminates, RNA chains, which, in turn, inhibits viral RNA production. Although evaluation of the potential effect of Remdesivir on SARS-Cov-2 and other coronaviruses has been considered consistent with therapeutic benefit, evidence from adequate clinical trials is missing ([45] and references therein). CQ and HCQ can cause serious toxicity and a thorough testing of their safety and efficacy is required before any off-label use is made of them for COVID-19 treatment.

## 9. Speculative Strategies with the Potential to Treat COVID-19 and New Variants

The scientific world has been making huge efforts to develop and improve vaccines and therapeutic treatments for COVID-19. It may be that a better appreciation of the subversion of the host cytoskeleton during CoV infections would help to inspire new strategies to control such infections and reduce CoV-related pathological damage. In the integrative sensor hypothesis, we have proposed that the cytoskeleton senses and integrates the general metabolic activity of the cell by binding metabolic enzymes and thereby changing its dynamics and its consumption of ATP and GTP. This binding would, in turn, depend on whether the enzyme catalyzes its cognate reaction [46,47]. A considerable body of evidence exists showing that some enzymes only bind to the cytoskeleton when it is active, whilst others only bind when inactive (for references see [46]). An innovative area of research might be to search for evidence of manipulation of cytoskeleton-binding metabolic enzymes by SAR-CoV-2. Such a search might include cytosine metabolism considering the special need that viruses have for it (which has led to several proposals for anti-viral strategies [48]), and the formation of cytoskeletal filaments by CTP synthase [49].

A very different type of approach to COVID-19 might be based on Defective Interfering Particles (DIPs), which occur in every family of viruses [50,51,52]. They contain degenerate virus genomes and cannot replicate on their own but need the functions of the parental virus. They have been found to interfere with the parental virus in ways that include virus replication, activation of immune responses, and promotion of virus persistence and can therefore influence the severity and spread of diseases [53]. Inspired by DIPs, it has long been thought that antiviruses or Therapeutic Interfering Particles might be constructed to combat many infectious diseases in both humans and animals [53]. In the case of SARS-CoV-2, a seductive possibility would be to construct such antiviruses to act not only on animal populations that harbor the virus (and related viruses) but also on human populations. In the latter case, it is conceivable that such DIPs exist already and are implicated in asymptomatic COVID-19.

The family of flaviviruses is one of the most medically important groups of arbovirus and comprises nearly 70 closely related RNA viruses. Flaviviruses can subvert the actin, MT, and IF networks of their host cells so as to facilitate entry, intracellular transport, replication, and exit. Favipiravir, known as T-705, is also a broad spectrum inhibitor of viral RNA polymerase that selectively and strongly inhibits the RNA-dependent RNA polymerase of RNA viruses, thereby preventing the synthesis of the viral RNA [54]. A major challenge for the widespread use of Remdesivir and Favipiravir is the development of resistance among CoVs; certain mutations in the RNA polymerase make the influenza A virus resistant to Favipiravir [55]. Both drugs are still used in the treatment of COVID-19 and it is therefore urgent to continue to evaluate and optimize them. Such optimization requires taking into account the different susceptibilities of the different human populations to these drugs and to the combinations of them with other treatments. It also requires a better understanding of the relationship between metabolism, cytoskeletal dynamics, and viral infections, a relationship that may extend to the microbiome [56].

## 10. Conclusions

It should be stressed that all three cytoskeletal networks are heavily involved in both physiological and pathological processes. Most of the data presented above concern the relationship between such processes and CoVs in general rather than SARS-CoV-2 in particular. Nevertheless, there are exciting implications for COVID-19 research in terms of the elucidation of the cytoskeletal structures and interactions required for infection and the identification of specific drug targets. More specifically, therapeutic goals might include directly targeting the ACE2 receptor and vimentin co-receptor (or indirectly targeting the ganglioside rafts containing these proteins), and perturbing cytoskeletal dynamics with anti-microtubule (anti-cancer) agents so as to limit damage whilst maintaining physiological functions.
